# Performance of clinical risk scores and prediction models to identify pathogenic germline variants in patients with advanced prostate cancer

**DOI:** 10.1007/s00345-023-04535-4

**Published:** 2023-08-01

**Authors:** Katharina Rebhan, Philipp D. Stelzer, Benjamin Pradere, Pawel Rajwa, Gero Kramer, Bernd Hofmann, Irene Resch, Ozan Yurdakul, Franco A. Laccone, Maria Gerykova Bujalkova, Mateja Smogavec, Yen Y. Tan, Robin Ristl, Shahrokh F. Shariat, Gerda Egger, Melanie R. Hassler

**Affiliations:** 1 Department of Urology, Comprehensive Cancer Center, Medical University of Vienna, Währinger Gürtel 18-20, 1090 Vienna, Austria; 2 Departments of Urology and Pediatric Urology, Klinik Ottakring, Vienna, Austria; 3 Department of Biomedical Imaging and Image-Guided Therapy, Medical University of Vienna, Vienna, Austria; 4 Department of Urology, La Croix Du Sud Hospital, Quint Fonsegrives, France; 5 Department of Urology, Medical University of Silesia, Zabrze, Poland; 6 Institute of Medical Genetics, Medical University of Vienna, Vienna, Austria; 7 Department of Obstetrics, Gynecology and Comprehensive Cancer Center, Medical University of Vienna, Vienna, Austria; 8 Center for Medical Statistics, Informatics and Intelligent Systems, Medical University of Vienna, Vienna, Austria; 9 Departments of Urology, Weill Cornell Medical College, New York, NY USA; 10 Department of Urology, University of Texas Southwestern, Dallas, TX USA; 11 Department of Urology, Second Faculty of Medicine, Charles University, Prague, Czech Republic; 12 Institute for Urology and Reproductive Health, I.M. Sechenov First Moscow State Medical University, Moscow, Russia; 13 Hourani Center for Applied Scientific Research, Al-Ahliyya Amman University, Amman, Jordan; 14 Karl Landsteiner Institute of Urology and Andrology, Vienna, Austria; 15 Department of Pathology, Medical University of Vienna, Vienna, Austria; 16 Ludwig Boltzmann Institute Applied Diagnostics, Vienna, Austria; 17 Comprehensive Cancer Center, Medical University of Vienna, Vienna, Austria

**Keywords:** Prostate cancer, Germline variants, DNA sequencing, Risk score, Genetic testing criteria, Family history

## Abstract

**Purpose:**

Determining the frequency and distribution of pathogenic germline variants (PGVs) in Austrian prostate cancer (PCa) patients and to assess the accuracy of different clinical risk scores to correctly predict PGVs.

**Methods:**

This cross-sectional study included 313 men with advanced PCa. A comprehensive personal and family history was obtained based on predefined questionnaires. Germline DNA sequencing was performed between 2019 and 2021 irrespective of family history, metastatic or castration status or age at diagnosis. Clinical risk scores for hereditary cancer syndromes were evaluated and a PCa-specific score was developed to assess the presence of PGVs.

**Results:**

PGV presence was associated with metastasis (*p* = 0.047) and castration resistance (*p* = 0.011), but not with personal cancer history or with relatives with any type of cancer. Clinical risk scores (Manchester score, PREMM5 score, Amsterdam II criteria or Johns Hopkins criteria) showed low sensitivities (3.3–20%) for assessing the probability of PGV presence. A score specifically designed for PCa patients stratifying patients into low- or high-risk regarding PGV probability, correctly classified all PGV carriers as high-risk, whereas a third of PCa patients without PGVs was classified as low risk of the presence of PGVs.

**Conclusion:**

Application of common clinical risk scores based on family history are not suitable to identify PCa patients with high PGV probabilities. A PCa-specific score stratified PCa patients into low- or high-risk of PGV presence with sufficient accuracy, and germline DNA sequencing may be omitted in patients with a low score. Further studies are needed to evaluate the score.

**Supplementary Information:**

The online version contains supplementary material available at 10.1007/s00345-023-04535-4.

## Introduction

Approximately 12% of patients with metastatic prostate cancer (PCa) harbor a PGV in genes involved in homologous DNA repair or mismatch repair such as *BRCA1*, *BRCA2*, *ATM*, *MSH2,* or other DNA damage repair (DDR) genes [1]. The presence of PGVs, in particular in *BRCA2*, is associated with a more aggressive disease behavior as well as a poorer prognosis, which is also reflected by a higher Gleason grade, a younger age, a more advanced disease stage at diagnosis, and lower survival rates [2–5]. While molecular profiling of localized PCa tumors with pathogenic *BRCA2* variants has shown an increased genomic instability and a mutational profile similar to metastatic disease [6], the impact of other PGVs on PCa is less well understood. Identification of PGV carriers with PCa is not only important for regular cancer screening or family cascade testing, but can offer the possibility for additional therapy options, such as PARP- or checkpoint inhibitors. Currently, several recommendations for germline testing in PCa patients are available, which take clinical features but also personal and family history into account [7–9]. For example, the European Association of Urology (EAU) PCa guideline from 2022 considers germline testing in men with metastatic PCa, men < 60 years with high-risk PCa, or family members with PCa [8]. However, this results in substantial overtesting. Currently, no available score predicts the presence of PGVs in PCa patients with positive family histories. For other tumor predisposition syndromes, such as hereditary breast and ovarian cancer (HBOC) caused by PGVs in *BRCA1/2* or for Lynch syndrome (LS) caused by DNA mismatch repair alterations, criteria and risk scores based on family history determine the probability of underlying PGVs and help select patients for germline testing. Examples comprise the Manchester scoring system, identifying patients with a > 10% probability of PGVs in *BRCA1/2* [10], the Amsterdam II criteria and the PREMM5 score, predicting the probability of underlying LS-associated gene alterations [11, 12], or the Johns Hopkins criteria to identify hereditary PCa families to offer early screening [13].

In summary, none of the existing scores or clinical criteria have been specifically evaluated in PCa patients. This study aimed to test the performance of these criteria and to develop a novel PCa-specific score based on personal and family cancer history to identify PCa patients with low or high probability for PGV presence.

## Materials and methods

### Patients

Patients with PCa treated at the Department of Urology of the Medical University of Vienna between 2019 and 2021 were offered germline genetic testing based on the National Comprehensive Cancer Network (NCCN) guidelines including high-grade localized or metastatic PCa [9]. Demographic and clinical data were collected from clinical documentation. Family history was patient-reported and obtained by questionnaires (Supplement 1 and 2). Patients were unselected regarding personal or family history and age at onset. All patients provided written informed consent and local ethics committee approved the study (EK-Nr: 1043/2020).

### Multi-gene panel sequencing and bioinformatics analysis

DNA extraction from EDTA-blood samples according to standard protocols as well as sequencing were performed at the Institute of Medical Genetics of the Medical University of Vienna. Multi-gene panel testing was enriched for genes associated with PCa by the custom designed „Prostate-Carcinoma-Panel V1 “ (PCa panel) from Illumina (San Diego, California, USA). The PCa panel included 25 genes: *ATM, ATR, BRCA1, BRCA2, BRIP1, CDH1, CHEK2, EPCAM, FAM175A (ABRAXAS1), FANCA, GEN1, MLH1, MLH3, MRE11A, MSH2, MSH6, NBN, PALB2, PMS2, PRAC2, PTEN, RAD51C, RAD51D, STK11* and *TP53*. This multi-gene panel was designed based on commonly used gene panels and frequently altered genes described in recent literature [1, 14–17].

Reported variants were reported as likely pathogenic and pathogenic variants or variants of uncertain significance (VUS) [18]. All reported variants were confirmed by Sanger sequencing (detailed in Supplement 3).

### Statistics and score evaluation

Descriptive statistics were used to analyze patient characteristics. Associations between PGVs and clinical features were examined using Chi-square and *t* test. *p* values < 0.05 (two-tailed) were considered significant. The Manchester score was calculated and counted positive if > 14 (unadjusted for tumor biology) [10]. Johns Hopkins criteria were fulfilled if the patient met one criteria [13]. Amsterdam II criteria were considered fulfilled if the patient met all criteria [11]. The PREMM5 score was calculated using the online platform (https://premm.dfci.harvard.edu/) and considered positive, if the overall predicted probability for LS was ≥ 2.5% (Tables S1–3) [12]. The individual scores were used to calculate sensitivity, specificity, positive predictive value (PPV), negative predictive value (NPV) and accuracy for presence of PGVs.

For PCa-specific score development, the variables were chosen based on clinical observations relevant for interpreting patients’ family pedigrees in routine genetic counseling. These variables comprised (i) personal history of gastrointestinal or male breast cancer, (ii) first-degree relatives with a history of gastrointestinal, breast, endometrial, ovarian or PCa, (iii) < 5 first-degree relatives with cancer histories available for assessment, (iv) personal history of cancer, and (v) second-degree relatives with history of gastrointestinal, breast, endometrial, ovarian or PCa (Table S4).

The variables were tested by logistic regression analysis, and variables with *p* values with < 0.15 and regression coefficients > 0.5 were considered significant for inclusion for further regression analysis [corresponding to variables (i), (ii), and (iii)]. They were included in a receiver operating characteristic (ROC)-curve to calculate the score’s cut-off. A cut-off of ≥ 0.917 resulted in highest sensitivity and specificity (Table S5).

To simplify the model, points for the PCa score were assigned by rounding the value of the regression coefficients for variables (i), (ii) and (iii) from the stepwise logistic regression to 1. The pretest probability for presence of PGVs was considered high if the score was ≥ 1.

All analyses were conducted with IBM SPSS-software version 23.

## Results

In this cross-sectional study, 313 men underwent germline genetic testing for 25 genes and were interviewed for personal and family history. The ethnicity was primarily Caucasian, the median age at PCa diagnosis was 64 years (42–83 years). At the time of enrollment, 78 patients had localized, non-metastatic PCa, and 235 had metastatic PCa, of whom 34.9% (*n* = 82) had de novo metastatic PCa. The majority of the patients were castration resistant (61.8%). In 30 patients (9.6%), an underlying PGV could be identified. Ethnicity, age, initial PSA values or ISUP grading at PCa diagnosis did not correlate with the occurrence of any PGVs, but the proportion of patients with metastasized or castration-resistant PCa was significantly higher in the group of patients with PGVs (*p* = 0.047, *p* = 0.011, respectively) (Table 1).

**Table 1:** Clinicopathological characteristics of the patients

Germline variants	Non-pathogenic (*n* = 283)	pathogenic (*n* = 30)	Total (*n* = 313)	*p* =
Age at diagnosis (median, IQR in years)	64 (58–70)	64 (53–68)	64 (58–70)	0.114
Ethnicity (*n*, relative frequency in %)				
Caucasian	236 (83.4%)	26 (86.7%)	262 (83.7%)	0.522
Asian	3 (1.1%)	0 (0%)	3 (1%)	
Hispanic	1 (0.4%)	0 (0%)	1 (0.3%)	
African	2 (0.7%)	1 (0.4%)	3 (1%)	
Unknown	41 (14.5%)	3 (10%)	44 (14.1%)	
ISUP Grade (*n*, relative frequency in %)				
1	16 (6%)	4 (13%)	20 (6.8%)	0.474
2	35 (13%)	2 (7%)	37 (12.5%)	
3	41 (15%)	6 (20%)	47 (15.9%)	
4	71 (27%)	7 (23%)	78 (26.4%)	
5	102 (38%)	11 (37%)	113 (38.3%)	
Unknown	18 (7%)	0 (0%)	18 (6.1%)	
Initial PSA (median, IQR ng/dl)	15 (7.6–57.5)	16 (6.9–40)	15 (7.5–55)	0.238
Stage of disease at germline testing (*n*, relative frequency in %)				
Metastasized	208 (73.5%)	27 (90%)	235 (75.1%)	**0.047**
Localized	75 (26.5%)	3 (10%)	78 (24.9%)	
Castration resistant	169 (59.7%)	25 (83.3%)	194 (62%)	**0.011**
Stage of disease at diagnosis (*n*, relative frequency in %)				
Localized (< pT3, N0, M0)	58 (20.5%)	8 (26.7)	66 (21.1%)	0.235
Locally advanced (≥ pT3 or N1)	119 (42%)	9 (30%)	128 (40.9%)	
De novo metastasized	71 (25.1%)	11 (36.7%)	82 (26.2%)	0.170
Unknown	35 (12.4%)	2 (6.7%)	37 (11.8%)	

Significance bold value is *p* < 0.05

*ISUP* international society of urological pathology, *PSA* prostate specific antigen, *IQR* interquartile range

**Fig. 1:**
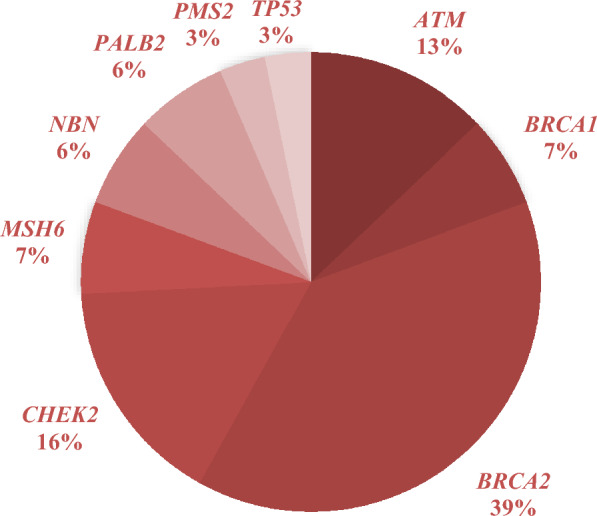
PGVs detected by germline testing in an Austrian advanced prostate cancer cohort. Percentages give percentage of individual PGVs among all PGV carriers (*n* = 30). Note that one patient carried PGVs in both *CHEK2* and *PALB2*

VUS were detected in 50 (16%) patients, and in 233 (74.4%) patients neither PGVs nor VUS were detected. The most frequent PGVs were found in *BRCA2* (*n* = 12), *CHEK2* (*n* = 5) and *ATM* (*n* = 4) (Fig. 1).

Regarding prior personal history, 54 patients (17.3%) had a personal history of at least one additional cancers, including basalioma, melanoma, colorectal carcinoma, urothelial carcinoma of the bladder, squamous cell carcinoma of the skin or upper tract urothelial cancer. Patients with or without PGVs did not display significant differences with regard to any personal cancer history (*p* = 0.708) (Table S6).

Most patients had at least one relative with any type of cancer diagnosis (74%, *n* = 234). In total, 68 relatives had been diagnosed with colorectal cancer, 60 with PCa, 52 with breast cancer and 20 with pancreatic cancer. There were no statistically significant differences regarding positive family history between patients with or without PGVs. Furthermore, the total number of relevant tumors (i.e., prostate, colon, upper urinary tract, pancreatic, breast, endometrial or ovarian cancer) (Table S7).

Next, we tested the performance of clinical criteria relying on positive family history for HBOC (Manchester score), LS-associated cancer (Amsterdam II criteria, PREMM5 score), or PCa (Johns Hopkins criteria) in indentifying PCa patients with high probabilities for underlying PGVs in *BRCA1/2* (*n* = 14) or DNA mismatch repair genes (*n* = 3). In the entire cohort, three patients (1%) had a positive Manchester score, and only one out of 14 patients with a PGV in *BRCA1/2* had a positive Manchester score. Ten patients (3.2%) fulfilled the Johns Hopkins criteria, and only one out of 30 patients with any PGV would have been considered at high risk of PGV presence by these criteria. 20 patients (6.4%) fulfilled the Amsterdam II criteria for LS. In our cohort, three patients had PGVs associated with LS, but Amsterdam II criteria identified none of these patients. Among the 19 patients for whom the PREMM5 score could be obtained, 6 patients (31.6%; 19% of total cohort) had a predicted probability for a PGV in LS-associated genes of ≥ 2.5%. One of the three patients with an LS-associated PGV fulfilled the PREMM5 testing criteria. In summary, all known scores had low sensitivities in detecting patients with PGVs in our PCa population (Table S8).

In genetic counselling, patients with either relevant personal or family history for specific cancer syndromes are at risk of PGV presence. Patients also should be evaluated for PGVs if their family history is too limited for assessment (i.e. less than 5 first-degree family members with cancer history available). Therefore, we defined variables relevant for PCa patients with PGVs (Table S4). Logistic regression and ROC-curve analysis were performed (Table S5). The final variables comprised personal history of gastrointestinal or male breast cancer, first-degree relatives with a history of gastrointestinal, breast, endometrial, ovarian or PCa, and < 5 first-degree relatives with cancer histories available for assessment. If any of these three variables is fulfilled, the pretest probability of PGVs is high (score ≥ 1), and the patient should undergo genetic testing (Table 2). Among all patients, 207 had a PCa risk score ≥ 1, including all 30 patients with PGVs. 106 patients had a score < 1 with low probability of PGVs, none of these patients tested positive for PGVs.

**Table 2:** A PCa-specific score to evaluate the risk of PGV presence

PCa-specific score	Points
Personal history of cancer: gastrointestinal (colon or pancreatic) or male breast cancer	1
First-degree relatives with history of gastrointestinal (colon or pancreatic), breast, endometrial, ovarian or PCa	1
< 5 first-degree relatives with cancer histories available for assessment (mother, father, brother, sister, son, daughter)	1

If any of the three items is present (score ≥ 1), the patient is considered at high risk of PGV presence

## Discussion

To our knowledge, this study reports results of the largest Austrian PCa population receiving germline sequencing in recent years. The prevalence of 9.6% for PGVs in advanced PCa is consistent with previous studies [1, 14, 19–21]. The most common PGVs were detected in *BRCA2* (3.8% of all patients; 40% of patients with PGVs), *CHEK2* (1.6%; 12.7%) and *ATM* (1.2%, 12.3%). A significantly higher prevalence of PGVs was found in patients with metastatic and/or castration-resistant PCa, in line with previous studies [2, 3, 5].

Regarding classic components of genetic assessment criteria such as personal or family cancer history, a direct comparison between patients with or without PGVs revealed neither a difference in the total number of diagnosed tumors, relatives with cancer, nor relevant tumors associated with HBOC or LS.

Noteworthy, there was also no difference considering only relatives with breast or ovarian cancer. This is in contrast to a recent study published by Sabol et al., reporting that a positive family history for breast or ovarian cancer was predictive for PGVs in their PCa cohort [22]. This discrepancy may be explained by a different emphasis on positive family history, which served as selection criterion for germline testing. In the study by Sabol et al., a large part of patients with localized PCa underwent germline testing due to positive family history. Approximately 13% of these patients with localized PCa harbored a PGV, in contrast to only 3.8% in our patient cohort.

Clinical criteria can be applied for other cancer entities within the HBOC or LS-tumor spectrum to better identify patients with a high probability for specific PGVs. In our PCa patient population, only a few patients with PGVs fulfilled the Amsterdam II criteria, the Manchester score criteria, or had a PREMM5 score ≥ 2.5 (0, 1, and 1 patient, respectively). These criteria were primarily designed to detect high probabilities for *BRCA1/2* or mismatch repair gene alterations in patients with breast, ovarian or colorectal cancer. In our patient population, these criteria had very low sensitivities to identify PCa patients with PGVs and thus seem to have no clinical value in this setting.

Current evidence points to personalized screening protocols and adjusted therapeutic management of PCa patients, with both germline and tumor sequencing impacting decision-making [23, 24]. However, performing genetic testing according to the current EAU guidelines would identify all metastatic patients with PGVs (*n* = 27), but with the disadvantage of overtesting in 88% (*n* = 208) of patients. We designed a PCa-specific score categorizing patients either as low or high probability for PGVs based on personal and family cancer history. Whereas all patients with PGVs had a positive score and would thus have a high probability of PGV presence, one third of the patients without PGVs had scores of < 1 with a low probability of PGV presence. Consequently, these patients may not need germline DNA sequencing.

Our study has limitations. With 313 PCa patients, sample size is modest. This study was not population-based. Furthermore, the population consists of mainly white Caucasian men, which could lead to selection bias as disparities in PGVs had been described among racial minorities [25, 26]. Evaluation of different, previously introduced scores relied on patients’ memory of personal and family history. Furthermore, each previous score was designed to detect certain PGVs in specific genes. For example, the PREMM5 model does not consider PCa as a LS-associated tumor entity and was not designed for the cases with absent LS associated cancers. Thus, this calculator was only applicable for a minority of PCa patients in this study (*n* = 17). Although the PCa-specific score showed promising value for giving a pretest probability for the presence of PGVs, it will need validation in a larger prospective cohort study.

## Conclusion

Current clinical criteria to identify patients at risk of PGVs are insufficient and warrant further research. A PCa-specific score based on personal and family history may stratify PCa patients into low- and high-risk groups for PGV presence and help simplifying diagnostic processes by excluding PCa patients with low pretest probabilities.

## Supplementary Information

Below is the link to the electronic supplementary material.

Supplementary file1 (PDF 102 KB)

Supplementary file2 (PDF 100 KB)

Supplementary file3 (DOCX 13 KB)

Supplementary file4 (DOCX 15 KB)

Supplementary file5 (DOCX 14 KB)

Supplementary file6 (DOCX 14 KB)

Supplementary file7 (DOCX 13 KB)

Supplementary file8 (DOCX 13 KB)

Supplementary file9 (DOCX 14 KB)

Supplementary file10 (DOCX 15 KB)

Supplementary file11 (DOCX 16 KB)

## Data Availability

Data are available upon request from Katharina Rebhan and Melanie R. Hassler, but access may be restricted due to Austrian law.
