# Supplementary material for: Performance of clinical risk scores and prediction models to identify pathogenic germline variants in patients with advanced prostate cancer
Source: World J Urol. 2023 Aug 1;41(8):2091–7. doi: 10.1007/s00345-023-04535-4 (PMC10415416; doi:10.1007/s00345-023-04535-4)
Supplement: Supplementary file 1 — Supplementary file1 (PDF 102 KB) [file 345_2023_4535_MOESM1_ESM.pdf]

Fragebogen genetisches Risikoprofil

Name, Geburtsdatum:
/Patientenetikett

Art der Krebserkrankung?	Alter bei Erstdiagnose (in Jahren)

Ihre ethnische Herkunft/Herkunft der Eltern:

___

Nehmen/nahmen Sie regelmäßige Untersuchungen zur Krebsvorsorge wahr (z.B.: PSA-Wert-Bestimmung, rektale Untersuchung, …)? ☐ JA ☐ NEIN

Falls ja, welche?

___

Falls ja, in welchem Alter haben Sie mit Vorsorgeuntersuchungen begonnen? _______________

Sind bei Ihnen bereits andere Krebserkrankungen aufgetreten? (Bitte denken Sie auch an stattgehabte Operationen/Eingriffe sowie deren Befunde z.B. Schilddrüse, Darm, auffällige Befunde der Haut, …) ☐ JA ☐ NEIN - Falls ja:

Um welche Krebserkrankung(en) handelt(e) es sich?	Alter bei Erstdiagnose (in Jahren)

Sind in Ihrer Familie Krebserkrankungen aufgetreten? (Bitte machen Sie Angaben zu allen Ihrer biologischen Verwandten, inkl. Anzahl der Geschwister, Kinder,… in Klammer. Bitte zutreffendes ankreuzen)

Gab/Gibt es eine Krebserkrankung bei Ihrer/m biologischen/m:	**J**A / **N**ein / **U**nbekannt (bitte ankreuzen)	Wenn ja, welche Krebserkrankung(en)?	Alter bei Erstdiagnose (in Jahren)
Mutter	J / N / U		
Vater	J / N / U		

Gab/Gibt es eine Krebserkrankung bei Ihrer/m biologischen/m:	**J**A / **N**ein / **U**nbekannt (bitte ankreuzen)	Wenn ja, welche Krebserkrankung(en)?	Alter bei Erstdiagnose (in Jahren)
Schwester(n) ( )	J / N / U		
Bruder/Brüder ( )	J / N / U		
Tochter/Töchter ( )	J / N / U		
Sohn/Söhne ( )	J / N / U		
Großmutter mütterlicherseits	J / N / U		
Großvater mütterlicherseits	J / N / U		
Großmutter väterlicherseits	J / N / U		
Großvater väterlicherseits	J / N / U		
Schwester/n Ihrer Mutter ( )	J / N / U		
Bruder/Brüder Ihrer Mutter ( )	J / N / U		
Schwester/n Ihres Vaters ( )	J / N / U		
Bruder/Brüder Ihres Vaters ( )	J / N / U		

Sind Krebserkrankungen bei weiteren Ihrer biologischen Verwandten aufgetreten?

☐ JA ☐ NEIN

Falls ja: (Bitte geben Sie den Verwandtschaftsgrad möglichst genau inkl. Angabe, ob mütter- oder väterlicherseits an)

Verwandtschaftsgrad:	Welche Krebserkrankung(en)?	Alter bei Erstdiagnose (in Jahren)
