# Supplementary material for: Performance of clinical risk scores and prediction models to identify pathogenic germline variants in patients with advanced prostate cancer
Source: World J Urol. 2023 Aug 1;41(8):2091–7. doi: 10.1007/s00345-023-04535-4 (PMC10415416; doi:10.1007/s00345-023-04535-4)
Supplement: Supplementary file 2 — Supplementary file2 (PDF 100 KB) [file 345_2023_4535_MOESM2_ESM.pdf]

UNIVERSITÄTSKLINIK FÜR UROLOGIE

MEDIZINISCHE UNIVERSITÄT WIEN
Allgemeines Krankenhaus der Stadt Wien

Genetic Risk Profile Questionnaire

Name, date of birth:
/patient label

Type of cancer?	Age at first diagnosis (in years)

Your ethnic background/origin or your parents' ethnic background/origin

____

Do/did you attend regular cancer screening examinations (e.g.: PSA level determination, rectal examination, ...)? ☐ YES ☐ NO

If yes, which ones?

____

If yes, at what age did you start screening?____

Have you already had other cancers? (Please also think of any surgeries/interventions you have had and their findings, e.g. thyroid gland, intestine, abnormal findings of the skin, ...)
☐ YES ☐ NO – If yes:

What cancer(s) are involved?	Age at first diagnosis (in years)

Have there been any cases of cancer in your family? (Please provide details of all your biological relatives, incl. number of siblings, children, ... in parentheses. Please tick as appropriate)

Was there a cancer in your biological:	**Y**ES / **N**o / **U**nknown (Please mark with a cross)	If yes, what type of cancer(s)	Age at first diagnosis (in years)
Mother	Y / N / U		
Father	Y / N / U		

UNIVERSITÄTSKLINIK FÜR UROLOGIE

MEDIZINISCHE UNIVERSITÄT WIEN   Allgemeines Krankenhaus der Stadt Wien

Was there a cancer in your biological:	**Y**ES / **N**o / **U**nknown (Please mark with a cross)	If yes, what type of cancer(s)	Age at first diagnosis (in years)
Sister(s) ( )	Y / N / U		
Brother(s) ( )	Y / N / U		
Daughter(s) ( )	Y / N / U		
Son(s) ( )	Y / N / U		
Grandmother maternal	Y / N / U		
Grandfather maternal	Y / N / U		
Grandmother paternal	Y / N / U		
Grandfather paternal	Y / N / U		
Your mother's sister(s) ( )	Y / N / U		
Your mother’s brother(s) ( )	Y / N / U		
Your father’s sister(s) ( )	Y / N / U		
Your father’s brother(s) ( )	J / N / U		

UNIVERSITÄTSKLINIK FÜR UROLOGIE

MEDIZINISCHE UNIVERSITÄT WIEN

Allgemeines Krankenhaus der Stadt Wien

Have cancers occurred in more of your biological relatives?

☐ YES ☐ NO

If yes: (Please state the degree of family relationship as precisely as possible, including whether maternal or paternal relative)

Degree of relationship:	What type of cancer(s)	Age at first diagnosis (in years)
