# Supplementary material for: Performance of clinical risk scores and prediction models to identify pathogenic germline variants in patients with advanced prostate cancer
Source: World J Urol. 2023 Aug 1;41(8):2091–7. doi: 10.1007/s00345-023-04535-4 (PMC10415416; doi:10.1007/s00345-023-04535-4)
Supplement: Supplementary file 3 — Supplementary file3 (DOCX 13 KB) [file 345_2023_4535_MOESM3_ESM.docx]

**Supplementary 3: Materials and Methods**

**Germline multi-gene panel sequencing and bioinformatics analysis**

DNA fragments were paired-end sequenced on an Illumina NextSeq 500 sequencing system. The obtained sequencing reads were aligned to the NCBI human genome assembly (hg19). Variant Calling was performed according to best practice guidelines (available at https://gatk.broadinstitute.org/hc/en-us) for calling single-nucleotide variants, insertions and deletions. The evaluation of the called variants was performed using VarSeq software from Golden Helix (Bozeman, Montana, USA).

Variants were ﬁltered based on minor allele frequency (MAF) using an in-house database including data from >1000 whole exomes and published disease-causing variants. Variants presented as heterozygous in more than 100 cases and/or homozygous in more than two cases in the internal database or in the Genome Aggregation Database (gnomAD) were ﬁltered out. We used six prediction tools for independent assessments of the pathogenicity of ﬁltered missense variants (SIFT, Polyphen2, HVAR, MutationTaster, MutationAssessor, FATHMM, FATHMM MKL Coding) and variants with at least three predictions as damaging/ pathogenic were selected. Loss-of-function variants were independently considered for further analysis. Databases such as ClinVar and Leiden Open Variation Database (LOVD) were assessed for further variant interpretation.

Furthermore, a copy number variation (CNV) analysis was performed for all samples comparing the calculated coverage of each sequenced sample to the existing coverage data obtained from BAM-ﬁles for previously analyzed in-house samples. This analysis was also performed by a module from VarSeq software from GoldenHelix. All reported CNVs were conﬁrmed by MLPA analysis.
