# Supplementary material for: Performance of clinical risk scores and prediction models to identify pathogenic germline variants in patients with advanced prostate cancer
Source: World J Urol. 2023 Aug 1;41(8):2091–7. doi: 10.1007/s00345-023-04535-4 (PMC10415416; doi:10.1007/s00345-023-04535-4)
Supplement: Supplementary file 4 — Supplementary file4 (DOCX 15 KB) [file 345_2023_4535_MOESM4_ESM.docx]

| **Cancer/gender** | **Age at diagnosis** | **Score** | **Calculation** |
| --- | --- | --- | --- |
| Female breast cancer | <30 | 11 |  |
|  | 30-39 | 8 |  |
|  | 40-49 | 6 |  |
|  | 50-59 | 4 |  |
|  | >59 | 2 |  |
| Male breast cancer | <60 | 13 |  |
|  | >59 | 10 |  |
| Ovarian cancer | <60 | 13 |  |
|  | >59 | 10 |  |
| Pancreatic cancer | Any age | 1 |  |
| Prostate cancer | <60 | 2 |  |
|  | >59 | 1 |  |
| **Total** |  |  |  |

**Table S1: Manchester Score:** A score is assigned for each family member with cancer based on tumor type and age at diagnosis. Maternal and paternal lineages should be assessed separately. Patients with a score >14 are eligible for germline *BRCA1/2* sequencing (adapted from [10]).
