# Supplementary material for: Performance of clinical risk scores and prediction models to identify pathogenic germline variants in patients with advanced prostate cancer
Source: World J Urol. 2023 Aug 1;41(8):2091–7. doi: 10.1007/s00345-023-04535-4 (PMC10415416; doi:10.1007/s00345-023-04535-4)
Supplement: Supplementary file 5 — Supplementary file5 (DOCX 14 KB) [file 345_2023_4535_MOESM5_ESM.docx]

| **Johns Hopkins criteria are fulfilled, if any of the following applies:** |
| --- |
| Are there three first degree relatives with prostate cancer (parents, siblings, children) including patient? |
| Are three generations affected by prostate cancer? E.g., paternal grandfather, paternal uncle and patient? |
| Are there two relatives who developed prostate cancer before the age of 55 (including the patient)? |

**Table S2: Johns Hopkins criteria:** Patients meeting any of the criteria would be regarded as hereditary prostate cancer patients (adapted from [13]).

13. Carter BS, Bova GS, Beaty TH, Steinberg GD, Childs B, Isaacs WB, et al. Hereditary prostate cancer: epidemiologic and clinical features. J Urol. 1993;150(3):797-802. doi: 10.1016/s0022-5347(17)35617-3.
