# Supplementary material for: Performance of clinical risk scores and prediction models to identify pathogenic germline variants in patients with advanced prostate cancer
Source: World J Urol. 2023 Aug 1;41(8):2091–7. doi: 10.1007/s00345-023-04535-4 (PMC10415416; doi:10.1007/s00345-023-04535-4)
Supplement: Supplementary file 6 — Supplementary file6 (DOCX 14 KB) [file 345_2023_4535_MOESM6_ESM.docx]

| **There should be at least 3 relatives with an HNPCC-associated cancer (colorectal, cancer of the endometrium, small bowel, ureter, or renal pelvis)** |
| --- |
| One should be a first-degree relative of the other two |
| At least 2 successive generations should be affected |
| At least 1 should be diagnosed before age 50 |
| Familial adenomatous polyposis should be excluded in the colorectal cancer case(s) if any tumors should be verified by pathological examination |

**Table S3: Amsterdam criteria II:** all criteria need to be fulfilled. HNPCC: hereditary nonpolyposis colorectal cancer (adapted from [11]).

11. Vasen HF, Watson P, Mecklin JP, Lynch HT. New clinical criteria for hereditary nonpolyposis colorectal cancer (HNPCC, Lynch syndrome) proposed by the International Collaborative group on HNPCC. Gastroenterology. 1999;116(6):1453-6. doi: 10.1016/s0016-5085(99)70510-x.
