# Supplementary material for: Performance of clinical risk scores and prediction models to identify pathogenic germline variants in patients with advanced prostate cancer
Source: World J Urol. 2023 Aug 1;41(8):2091–7. doi: 10.1007/s00345-023-04535-4 (PMC10415416; doi:10.1007/s00345-023-04535-4)
Supplement: Supplementary file 7 — Supplementary file7 (DOCX 13 KB) [file 345_2023_4535_MOESM7_ESM.docx]

| **Variables for PCa-associated score** | **Regression coefficient** |  | **p-value** |
| --- | --- | --- | --- |
| i) Personal history of cancer: gastrointestinal (colon or pancreatic) or male breast cancer | 1.220186 |  | 0.10887 |
| ii) First-degree relatives with history of gastrointestinal (colon or pancreatic), breast, endometrial, ovarian or PCa | 1.114182 |  | 0.00659 |
| iii) <5 first-degree relatives with cancer histories available for assessment (mother, father, brother, sister, son, daughter) | 0.929606 |  | 0.02008 |
| iv) Personal history of cancer: other cancer | -0.005835 |  | 0.99173 |
| v) Second-degree relatives with history of gastrointestinal (colon or pancreatic), breast, endometrial, ovarian or PCa | -0.444799 |  | 0.39943 |

**Table S4: Logistic regression for variables relevant for interpreting patients’ family pedigrees.** Variables with regression coefficients >0.5 and a p-value <0.15 were included in further logistic regression and in ROC-curve analysis to calculate a cut-off for a score.
