# Supplementary material for: Performance of clinical risk scores and prediction models to identify pathogenic germline variants in patients with advanced prostate cancer
Source: World J Urol. 2023 Aug 1;41(8):2091–7. doi: 10.1007/s00345-023-04535-4 (PMC10415416; doi:10.1007/s00345-023-04535-4)
Supplement: Supplementary file 8 — Supplementary file8 (DOCX 13 KB) [file 345_2023_4535_MOESM8_ESM.docx]

| **Variables for PCa-associated score** | **Regression coefficient** |  | **p-value** |
| --- | --- | --- | --- |
| i) Personal history of cancer: gastrointestinal (colon or pancreatic) or male breast cancer | 1.2237 |  | 0.09537 |
| ii) First-degree relatives with history of gastrointestinal (colon or pancreatic), breast, endometrial, ovarian or PCa | 1.0658 |  | 0.00853 |
| iii) <5 first-degree relatives with cancer histories available for assessment (mother, father, brother, sister, son, daughter) | 0.9172 |  | 0.02117 |

**Table S5: Logistic regression and regression coefficients for selected variables from Table S4.** Significant variables (p<0.1) were included in a receiver operating characteristic (ROC)-curve to calculate the score’s cut-off. A cut-off of ≥0.917 resulted in highest sensitivity and specificity (0.93 and 0.35, respectively).
