# Supplementary material for: Performance of clinical risk scores and prediction models to identify pathogenic germline variants in patients with advanced prostate cancer
Source: World J Urol. 2023 Aug 1;41(8):2091–7. doi: 10.1007/s00345-023-04535-4 (PMC10415416; doi:10.1007/s00345-023-04535-4)
Supplement: Supplementary file 9 — Supplementary file9 (DOCX 14 KB) [file 345_2023_4535_MOESM9_ESM.docx]

| **Germline variant** | **Non-pathogenic (n=283)** | **pathogenic (n=30)** | **Total (n=313)** | **p=** |
| --- | --- | --- | --- | --- |
| Any other tumor (n, frequency in %) | 48 (17.3%) | 6 (20%) | 54 (17.5%) | 0.708 |
| None | 230 (82.7%) | 24 (80%) | 254 (82.5%) |  |
| Unknown | 4 | 1 | 5 |  |
| Total number of other tumors (mean, SD) | 1.2 (0.5) | 1.3 (0.8) | 1.2 (0.5) | 0.394 |
| Number of relevant tumors (mean, SD) | 1 (0.2) | 1.1 (0.3) | 1 (0.2) | 0.137 |
| Tumor entities | | | | |
| Melanoma | 10 | 3 | 13 |  |
| Breast cancer | 0 | 1 | 1 |  |
| Pancreatic cancer | 1 | 0 | 0 |  |
| Colorectal cancer | 7 | 4 | 11 |  |
| Other tumor entity (Number of patients) | 23 | 7 | 30 |  |

**Table S6: Personal cancer history of patient cohort:** One patient without a PGV had a prior diagnosis of melanoma and of colorectal cancer. Colon, upper urinary tract, pancreatic or breast cancer were regarded as tumor predisposition syndrome-associated relevant tumors. SD denotes standard deviation.
