# Supplementary material for: Performance of clinical risk scores and prediction models to identify pathogenic germline variants in patients with advanced prostate cancer
Source: World J Urol. 2023 Aug 1;41(8):2091–7. doi: 10.1007/s00345-023-04535-4 (PMC10415416; doi:10.1007/s00345-023-04535-4)
Supplement: Supplementary file 10 — Supplementary file10 (DOCX 15 KB) [file 345_2023_4535_MOESM10_ESM.docx]

|  | **No-PGVs** | | **PGVs** | | **All** | |  |
| --- | --- | --- | --- | --- | --- | --- | --- |
|  | **Frequency** | **Relative Frequency** | **Frequency** | **Relative Frequency** | **Frequency** | **Relative Frequency** | **p-value Chi² Test** |
| FDR with colorectal cancer | 26 | 9.2% | 5 | 16.7% | 31 | 9.9% | 0.192 |
| no FDR with colorectal cancer | 257 | 90.8% | 25 | 83.3% | 282 | 90.1% |  |
| FDR with Breast cancer | 31 | 11.0% | 6 | 20.0% | 37 | 11.8% | 0.145 |
| no FDR with Breast cancer | 252 | 89.0% | 24 | 80.0% | 276 | 88.2% |  |
| FDR with ovarian cancer | 6 | 2.1% | 1 | 3.3% | 7 | 2.2% | 0.669 |
| no FDR with ovarian cancer | 277 | 97.9% | 29 | 96.7% | 306 | 97.8% |  |
| FDR with pancreatic cancer | 11 | 3.9% | 1 | 3.3% | 12 | 3.8% | 0.881 |
| no FDR with pancreatic cancer | 272 | 96.1% | 29 | 96.7% | 301 | 96.2% |  |
| FDR with P | 33 | 11.7% | 5 | 16.7% | 38 | 12.1% | 0.425 |
| no FDR with PCa | 250 | 88.3% | 25 | 83.3% | 275 | 87.9% |  |

**Table S7: PGVs in first-degree relatives:** PGV: pathogenic germline variant**,** FDR: first-degree relative, PCa: Prostate cancer
