# Supplementary material for: Performance of clinical risk scores and prediction models to identify pathogenic germline variants in patients with advanced prostate cancer
Source: World J Urol. 2023 Aug 1;41(8):2091–7. doi: 10.1007/s00345-023-04535-4 (PMC10415416; doi:10.1007/s00345-023-04535-4)
Supplement: Supplementary file 11 — Supplementary file11 (DOCX 16 KB) [file 345_2023_4535_MOESM11_ESM.docx]

| Diagnostic accuracy | all patients (N=313) | True positives (N=) | False negatives (N=) |
| --- | --- | --- | --- |
| Johns Hopkins criteria (fulfilled in 10 patients) | | 1 | 29 |
| Sensitivity | 3.3% |  |  |
| Specificity | 96.8% |  |  |
| PPV | 10.0% |  |  |
| NPV | 90.4% |  |  |
| Accuracy | 87.9% |  |  |
| Manchester score (fulfilled in 3 patients) | | 1 | 13 |
| Sensitivity | 3.3% |  |  |
| Specificity | 99.3% |  |  |
| PPV | 33.3% |  |  |
| NPV | 90.6% |  |  |
| Accuracy | 90.1% |  |  |
| Amsterdam II criteria (fulfilled in 20 patients) | | 0 | 3 |
| Sensitivity | 20.0% |  |  |
| Specificity | 95.1% |  |  |
| PPV | 30.0% |  |  |
| NPV | 91.8% |  |  |
| Accuracy | 87.9% |  |  |
| PREMM5 score criteria (fulfilled in 6 patients) | | 1 | 2 |
| Sensitivity | 6.7% |  |  |
| Specificity | 98.6% |  |  |
| PPV | 33.3% |  |  |
| NPV | 90.9% |  |  |
| Accuracy | 89.8% |  |  |
| PCa-risk score (fulfilled in 208 patients) | | 30 | 0 |
| Sensitivity | 100% |  |  |
| Specificity | 37.2% |  |  |
| PPV | 14.5% |  |  |
| NPV | 100% |  |  |
| Accuracy | 43.3% |  |  |

**Table S8: Cross tables analyzing diagnostic accuracy of clinical scores and criteria:** PPV denotes positive predictive value and NPV negative predictive value. The true positive rate refers to the associated PGV for each criterion (i.e., PGV in *BRCA1/2* for Manchester Criteria and in LS-associated genes for Amsterdam II and PREMM5 score).
